# Allogeneic Stem Cell Transplant for Myelofibrosis and Myelodysplastic Syndromes: A Contemporary Review

**DOI:** 10.1002/ajh.27660

**Published:** 2025-03-13

**Authors:** Nico Gagelmann, Nicolaus Kröger

**Affiliations:** ^1^ Department of Stem Cell Transplantation University Medical Center Hamburg‐Eppendorf Hamburg Germany

**Keywords:** busulfan, myelofibrosis, transplantation, treosulfan

## Abstract

Allogeneic hematopoietic stem‐cell transplantation (HCT) remains the only potentially curative therapy for patients with myelodysplastic neoplasms (MDS) and myelofibrosis (MF) and is the standard care for eligible patients with higher‐risk disease. Despite significant advancements, both diseases pose unique challenges due to their clinical and molecular heterogeneity, necessitating personalized approaches to patient selection, timing, and transplant management. For MDS, genomic profiling has revolutionized prognostic frameworks such as IPSS‐M, enabling tailored therapeutic decisions. In MF, driver mutations (e.g., *JAK2, CALR, MPL*) and additional high‐risk molecular markers provide critical insights into disease biology and transplant outcomes. Optimal timing of HCT is critical, and recent models might help personalize treatment approaches. Molecular measurable residual disease monitoring has demonstrated prognostic value in both diseases, guiding preemptive strategies to mitigate relapse risk. Harnessing molecular technologies, clinical expertise, patient‐centered decision‐making, and innovative pharmaceutical strategies offers an exciting opportunity to shape a transformative and curative treatment framework. Here, we provide a contemporary review on HCT for MDS and MF, highlighting up‐to‐date insights into disease biology, standard of care, and recommendations, as well as open avenues.

## Graphical abstract

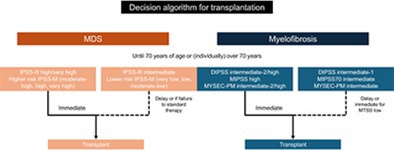

## 1 Introduction

Allogeneic hematopoietic stem‐cell transplantation (HCT) is a pivotal treatment option in the management of myeloproliferative neoplasms (MPN) and myelodysplastic syndromes (MDSs), two groups of hematologic disorders characterized by abnormal blood cell production and marrow dysfunction [1]. While both diseases have seen advances in drug development, transplantation is a potentially curative therapy and has become the standard care of treatment for eligible patients with higher‐risk myelofibrosis (MF) and MDS [2, 3].

In MDS, where the bone marrow fails to produce enough healthy blood cells and patients often suffer from severe anemia, infections, and bleeding disorders, HCT offers an option for disease control and cure. This is, particularly, important in higher‐risk MDS, where the likelihood of progression to acute myeloid leukemia (AML) is high, and limited conventional treatments may only offer temporary symptom relief and limited survival benefits without the potential to cure the disease [4].

The hallmark of MPN, namely MF (primary or secondary evolving from polycythemia vera or essential thrombocythemia), is driver mutations (*JAK2, CALR*, and *MPL*) present in ~90% of patients, progressing bone marrow fibrosis, anemia, and splenomegaly. For MF, transplantation can halt disease progression, even resolve bone marrow fibrosis, clear driver mutation burdens completely, and improve survival rates, especially in higher‐risk disease [5]. The main challenges are clinical heterogeneity, relative older age and consequently increased comorbidity burden, as well as transplant complications such as graft‐versus‐host disease (GVHD) [6], making nuanced and dedicated HCT approaches more relevant than ever.

Both MDS and MF share a clinical and mutational variety that is associated with heterogeneous outcomes, making the decision for such an intensive treatment like HCT especially challenging. Here, we give a contemporary overview of the current status and open avenues for HCT in these two entities.

## 2 Evolution and Current Outcomes of Transplantation in MDS and MF

**FIGURE 1:**
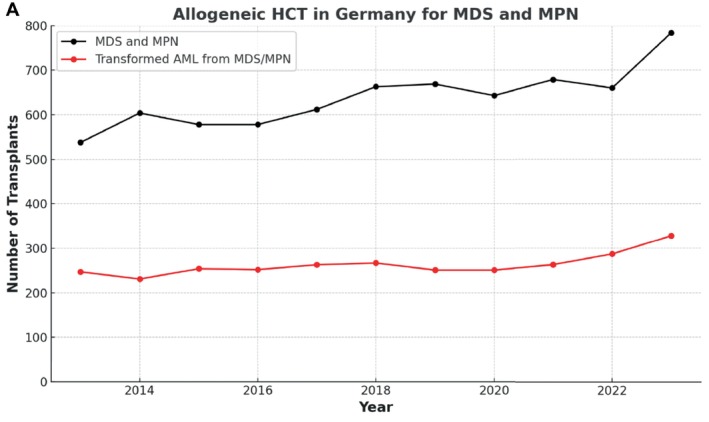
Transplant numbers in Germany for MDS and MPN.

In the last decade, more than 20 000 HCT have been performed for MDS and MPN in Europe, with Germany accounting for one‐third, treating approximately 7000 patients with MDS and MPN, reflecting an increase of two‐thirds over one decade [7, 8]. Numbers of HCTs steadily increased for MDS and MPN as well as for patients with transformed AML (Figure 1). The median age of patients undergoing transplantation has significantly increased, with nearly one‐third aged more than 65 years. The use of haploidentical donors grew substantially, while the numbers of matched‐sibling donors steadily decreased over time, and peripheral blood has become the primary source of stem cells. Reduced‐intensity conditioning (RIC) remains the most utilized HCT strategy, especially in MF, aligning with the higher median age of transplant recipients [9].

For MDS, the 5‐year overall survival (OS) rate posttransplant is approximately 60%–70%, with relapse rates ranging from 20% to 30% [10, 11]. Non‐relapse mortality (NRM) has improved with advancements in supportive care but remains a considerable risk, particularly, in older patients or those with comorbidities, being 10%–30% [12, 13]. For MF, specifically MF, the 5‐year OS is around 60% in large centers, with a 1‐year relapse incidence of approximately 10%–20% and a NRM of 20%–30% [14, 15, 16]. Long‐term outcomes, including OS, progression‐free survival (PFS), NRM, and relapse rates, show progressive improvement, probably attributed to improved patient selection, enhanced infection management (especially CMV prophylaxis) [17], optimized conditioning protocols, and better supportive care.

## 3 Decision for Transplantation in MDS

### 3.1 Role of Disease Biology and Classification

In MDS, the advent of genomic profiling has fundamentally transformed the diagnostic and prognostic approach, moving beyond traditional reliance on morphologic abnormalities to a molecular focus on clonal hematopoiesis and specific genetic lesions linked to clinical outcomes [18]. Next‐generation sequencing (NGS) is now recommended for all newly diagnosed MDS patients, performed using peripheral blood or bone marrow samples, with gene panels tailored to include markers relevant for the Molecular International Prognostic Scoring System (IPSS‐M) and germline predisposition [19, 20, 21]. Cytogenetics remains a critical parallel assessment, and *TP53* mutations require detailed evaluation of allelic state, deletions, and copy number variations [22]. Recent classification systems, including the WHO Fifth Edition and ICC [23], integrate genetic criteria alongside morphology to define MDS subtypes and revise traditional thresholds for AML diagnosis, incorporating cases with specific genetic features, regardless of blast count. Comprehensive bone marrow analysis encompassing morphology, karyotyping, and sequencing is essential, with diagnostic panels targeting mutations like *SF3B1* and *TP53*, while larger panels are mandatory for prognostic purposes under IPSS‐M.

The IPSS‐M advances risk stratification by integrating somatic mutations, outperforming the IPSS‐R in predicting survival and posttransplant outcomes, particularly, in untreated patients [24, 25]. Molecular profiling has further delineated 18 genetic subtypes, enabling more precise therapeutic and transplant decisions. For example, patients with *DDX41* mutations require specific donor selection strategies, while those with *TP53*‐complex MDS benefit from clinical trial enrollment due to poor transplant outcomes. Germline predisposition to MDS, found in up to 10% of adult patients undergoing HCT, highlights the need for genetic counseling, careful donor selection, and tailored conditioning regimens.

### 3.2 Timing of Transplantation and Patient Selection

Several decision models aimed at identifying the right timepoint for HCT in MDS reflect the evolution of risk stratification (from IPSS to IPSS‐M) and understanding of the disease. In addition, two important prospective trials compared outcomes of MDS for HCT and non‐HCT approaches.

The first decision report from 20 years ago showed delayed HCT maximizes survival for low and intermediate‐1 IPSS [26], especially in patients under 40. For intermediate‐2 and high‐risk groups, immediate transplantation at diagnosis yields the best outcomes. Quality‐of‐life adjustments did not alter these optimal strategies. Another Markov model showed that delaying HCT to intermediate IPSS‐R risk increases life expectancy, while late‐stage transplants reduce it [27]. Switching from IPSS to IPSS‐R improved decisions in 29% of cases, adding 2 years of life expectancy. In advanced stages, hypomethylating agents (HMAs) before transplant boosted survival, especially in older patients, optimizing MDS treatment outcomes. The most recent decision analysis showed that an IPSS‐M‐based policy suggested delayed HCT for low and moderate‐low risk MDS [24], while immediate HCT benefits moderately high, high, and very high‐risk groups. Transitioning from IPSS‐R to IPSS‐M altered HCT strategies for one‐third of patients, leading to a significant life expectancy gain.

Two prospective biology‐assignment trials have demonstrated the superiority of RIC‐ and matched‐donor‐based HCT over non‐transplant approaches [28, 29]. In an intention‐to‐treat analysis of the US trial of 309 patients [29], the adjusted 3‐year OS was significantly higher in the donor arm (compared to the no‐donor arm [48% vs. 27%; *p* < 0.001]). Leukemia‐free survival at 3 years was also greater in the donor arm (36% vs. 21%; *p* = 0.003). The survival benefit was consistent across all subgroups.

In the second German trial of 190 patients which compared HCT following azacytidine pretreatment with continuous azacytidine treatment [28], 28 had to be excluded for various reasons, and of the 162 patients who began azacytidine induction, only 108 (67%) progressed to treatment allocation (81 to HCT and 27 to continued azacytidine). Importantly, dropouts were due to disease progression (*n* = 26), death (*n* = 12), or other reasons (*n* = 16), with 7% dying during 5‐aza treatment before allocation. The 1‐year cumulative NRM was 19% and the 3‐year event‐free survival and OS were significantly better in the HCT group compared to azacytidine (34% and 50% vs. 0% and 32%). Of note, among 14 patients who progressed on 5‐aza and received salvage HCT, 43% were alive at last follow‐up. In conclusion, a matched donor and RIC‐based HCT conferred a significant survival advantage in older, fit patients with higher‐risk MDS, while bridging with 5‐aza is associated with substantial dropouts due to progression, mortality, and adverse events.

The aforementioned advances underscore the critical role of nuanced decision making using genomic insights together with disease‐, patient‐, and transplant‐related factors, offering potential for precision medicine approaches and improved clinical outcomes.

Assessing patient eligibility for HCT in MDS involves considering disease‐related and patient‐related risk factors, donor availability, and patient preferences. Disease‐related factors include genomic profiling for somatic mutations, risk stratification using IPSS‐M (if possible), and clinical manifestations such as cytopenias or leukemic transformation risk. Higher‐risk MDS patients (with a median survival of ~1.5 years) are typically candidates for immediate HCT, while individual balancing of risks and benefits for lower‐risk patients is needed, with factors like transfusion dependence or germline predisposition influencing decisions. Patient‐related factors focus on age, performance status (Karnofsky Performance Status ≥ 80), comorbidities, frailty assessments, and prior conditions such as iron overload, which may necessitate chelation therapy. Age is not an a priori contraindication for considering HCT [30].

In cases of *TP53*, a recent analysis within a prospective clinical trial highlighted the importance of immediate HCT in this very high‐risk group [31]. In patients with IPSS‐M very high risk but no TP53 mutation, OS was dramatically better if a donor was available (68% vs. 0% at 3 years; *p* = 0.001). In conclusion, HCT improved OS for patients with *TP53* mutations regardless of allelic status and provided favorable outcomes for IPSS‐M very high‐risk patients without *TP53* mutations when a donor was available. Other previous reports suggested the particular effectiveness of HCT for patients with mutated *TP53* or complex karyotype alone [32].

**FIGURE 2:**
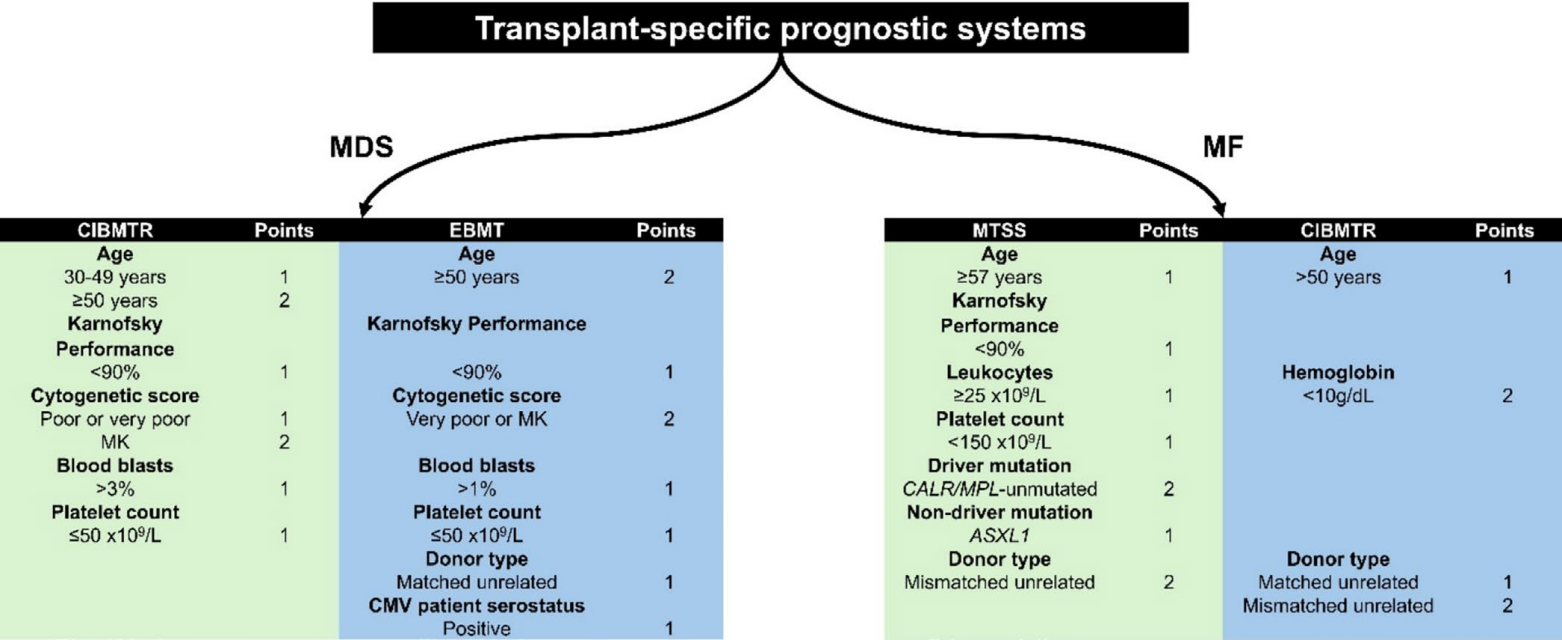
Transplant‐specific scorings systems for MDS and MPN.

This finding underscores that the timing of HCT should be guided by disease severity, but also by HCT‐specific factors (Figure 2).

## 4 Decision for Transplantation in MF

### 4.1 Role of Disease Biology and Classification

MPMs, in particular MF, are complex clonal hematopoietic malignancies characterized by molecular and clinical heterogeneity. At the molecular level, driver mutations in *JAK2*, *CALR*, and *MPL* are prevalent in approximately 90% of patients and define the disease's pathogenesis, promoting dysregulated JAK‐STAT signaling and cytokine overproduction [33]. In addition, high‐risk somatic mutations (e.g., *ASXL1, EZH2, IDH1, IDH2, SRSF2*, and *U2AF1Q157*) are associated with adverse outcomes, including reduced survival and increased leukemic transformation risk, classifying them as high‐molecular‐risk mutations (HMR) [34].

Traditional risk classification based on clinical and cytogenetic features only, reflected in the DIPSS and DIPSS‐plus [35, 36]. In the molecular era of MF, advanced prognostic tools for primary MF have been developed, leveraging the aforementioned HMR profile. Prognostic systems such as MIPSS70 and its updated versions (MIPSS70‐plus and MIPSS70‐plus v2.0) integrate these molecular markers, refined anemia thresholds, and cytogenetic risk classifications [37]. For secondary MF, the MYSEC‐PM stratifies patients into four risk categories with distinct survival outcomes [38].

Another hallmark feature of MF is splenomegaly and plays a significant role in the disease's biology and clinical manifestations. Despite being included as a response assessment in prospective trials [39], splenomegaly is not regularly included in current prognostic systems (possibly due to lack of retrospective data and harmonized assessment). It arises due to extramedullary hematopoiesis, a compensatory response to bone marrow failure caused by progressive fibrosis [40]. The spleen becomes a site of aberrant hematopoiesis, contributing to its massive enlargement, which often leads to symptomatic splenomegaly, including abdominal discomfort, early satiety, and left upper quadrant pain. Splenomegaly is also associated with heightened inflammatory cytokine production, further exacerbating systemic symptoms such as fatigue, fever, and weight loss. From a molecular perspective, splenomegaly may reflect disease progression and a higher burden of clonal hematopoiesis, often associated with high‐risk mutations, such as those in *ASXL1* or *SRSF2* [41, 42]. Additionally, splenomegaly poses therapeutic challenges, as it can impair drug delivery, increase transfusion requirements, and complicate hematopoietic cell transplantation by increasing the risk of engraftment failure or complications [43].

### 4.2 Timing of Transplantation and Patient Selection

Determining the optimal timing for HCT in patients with MF is challenging, particularly, without prospective clinical trial data. A recent Markov model suggested that the best time for HCT depends on the patient's risk category (although here only clinical features were used) and calculating life expectancy and progression risk [44]. For patients with DIPSS high‐risk disease, the peak benefit in terms of survival occurs 10 months after diagnosis, while for intermediate‐2‐risk patients, the peak is at 17 months. Intermediate‐1‐risk patients experience a delayed benefit, with peak survival gain at 21 months, while low‐risk patients achieve the greatest net gain in life expectancy after 29–45 months.

One pivotal retrospective study a decade ago determined the indication for HCT in MF using the DIPSS classification [45]. This study, using left‐truncation to account for attrition bias when comparing HCT and non‐HCT approaches, showed significantly improved OS for HCT in DIPSS intermediate‐2 and high risk, while patients with low risk clearly benefited from non‐HCT approaches. The majority of patients with intermediate‐1 risk showed benefit from non‐HCT approaches, while a subgroup of patients experienced excellent and improved outcomes with HCT. This study was confirmed by the CIBMTR [46]. In the era of molecular classification, a most recent study in primary MF showed that HCT improves survival in high‐risk MIPSS70 and high/very high‐risk MIPSS70 + V2 patients, while survival benefits for intermediate MIPSS70 patients occur only with low/intermediate HCT‐specific risk (see below) [47]. Overall, while HCT increases first‐year mortality, it provides significant survival benefits beyond 1 year for higher‐risk patients, confirming that optimal timing of HCT necessitates the combined use of patient‐, disease‐, and HCT‐related factors.

Additionally, most receive a JAK inhibitor, such as ruxolitinib. A response model based on factors like spleen size reduction, red blood cell transfusions, and dosage after 6 months of ruxolitinib therapy (RR6) has been shown to predict poor prognosis [48]. Patients with a poor response (characterized by insufficient spleen reduction and a higher transfusion need) should be evaluated for transplantation. The RR6 model has been validated, confirming its utility in identifying candidates for HSCT [49, 50]. However, the RR6 showed inferior performance in discriminating lower‐risk patients and appeared to be affected by underlying mutational status. Based on these findings, transplant‐eligible patients on JAK inhibitors should undergo systematic response assessments, with those in the high‐risk RR6 category considered for HSCT after 6 months of treatment.

The Myelofibrosis Transplant Scoring System (MTSS) incorporates not only clinical and molecular but also transplant‐specific factors like donor source and the Karnofsky Performance Score to predict outcomes posttransplant [14]. Retrospective studies indicated that patients with DIPSS intermediate‐2 or high risk, and more recently MIPSS70 high risk, MYSEC‐PM high or intermediate‐2 risk, and MTSS low or intermediate risk are optimal candidates for HCT [45]. Additionally, those with intermediate‐risk scores (DIPSS or MIPSS70) may also be considered, factoring in patient preferences, clinical trial availability, and *TP53* mutational status.

As with MDS, patients with *TP53* multihit configuration present a very high‐risk group due to poor outcomes and high leukemic transformation rates [51, 52]. Although evidence is not as strong as for MDS, recent studies suggested a global benefit of HCT over non‐transplant approaches for all *TP53* patients, if eligible.

As with MDS, age per se should not be a contraindication. Over the past decade, HCT numbers have increased significantly, particularly, since 2019, with improved patient comorbidities and a preference for RIC‐based HCT [53]. The 3‐year OS was 55%, with a 1‐year cumulative incidence of relapse at 7% and NRM at 22%. The 3‐year GVHD‐ and relapse‐free survival was 37%. Among variables analyzed, the non‐*CALR*/*MPL* genotype was independently associated with better survival, while the comorbidity index and conditioning intensity did not significantly influence outcomes. This study demonstrated the feasibility and curative potential of HCT in patients aged 70 or older with MF, highlighting improved fitness and increasing HCT utilization in this population. Thus, in individuals older than 70 years, HCT may be offered based on good performance status, HLA‐matched donors, and a balance of disease‐ and patient‐specific features, providing a personalized approach to treatment decisions [2, 53].

## 5 How to Perform Transplantation in MDS and MF in 2025

### 5.1 Donor Selection

In MDS, the current hierarchy of donor selection recent advances in donor selection have been shaped by an increased use of haploidentical donors, the application of posttransplant cyclophosphamide (PTCY), and emerging insights into the role of donor age and HLA matching. Emerging evidence suggests prioritizing haploidentical donors when HLA‐matched relatives or unrelated donors (MUDs) are unavailable, while umbilical cord blood should only be considered as a last resort [54]. Findings from the CIBMTR indicated that OS in MDS receiving allografts from haploidentical donors and MUDs is comparable, despite higher relapse rates with haploidentical donors, attributed to increased chronic GVHD rates with MUDs [55], while results from the EBMT showed rather dismal outcomes [56]. Parental donors in haploidentical transplants were associated with worse outcomes compared to sibling donors [57].

Donor age is increasingly recognized as a critical factor influencing the sequence of donor type selection, with HLA‐matched relatives aged over 60 years being considered less optimal. Data from the CIBMTR suggest that younger unrelated donors may yield superior outcomes compared to older HLA‐matched relatives (in the context of PTCY) [58].

In MF, the CIBMTR reported a decade ago on HCT outcomes based on donor type. The 5‐year OS was 56% for patients with HLA‐matched‐sibling donors and 48% for those with HLA‐matched unrelated donors, with a fourfold higher NRM in the latter group, establishing matched sibling donors as gold standards, if available [59]. Further multicenter studies in the contemporary era showed similar outcomes between matched‐siblings and MUDs [14, 60]. Alternative donor options have been less studied. Another multicenter retrospective study of 69 patients undergoing haploidentical HCT with PTCY reported a 3‐year OS of 72%, relapse‐free survival of 44%, and a NRM of 23% [61, 62]. Another CIBMTR study evaluated the impact of donor types on HCT outcomes in 1597 MF patients (transplanted between 2013 and 2019). The use of haploidentical donors increased from 3% in 2013 to 19% in 2019. Matched‐sibling donor HCTs showed superior OS within the first 3 months, with significantly lower graft failure rates compared to haploidentical, MUDs, and mismatched unrelated donor transplants [63].

In conclusion, matched‐sibling donors remain the preferred donor source for HCT unless donor age or comorbidities preclude their use. In the absence of an HLA‐matched‐sibling or unrelated donor, haploidentical HSCT and 7/8 HLA‐matched unrelated donors offer at least comparable outcomes, while cord blood transplantation is generally not recommended for MDS and MF patients.

## 6 Conditioning and GVHD Prophylaxis

The choice of conditioning regimen and GVHD prophylaxis in HCT for MDS and MF alike depends on patient‐specific factors such as age, performance status, and disease risk. We summarized current evidence on comparisons of different conditioning intensities in both entities in Table 1 [64, 65, 66, 67, 68, 69, 70, 71, 72, 73].

**TABLE 1:** Results of different conditioning intensities in MDS and myelofibrosis.

Study	Disease	Regimen	OS	NRM	Relapse
Myelofibrosis					
McLornan et al.		RIC vs. MAC	3 years: 53% vs. 51%	32% vs. 33%	17% vs. 20%
Gagelmann et al.		RIC vs. MAC	6 years: 63% vs. 59%	26% vs. 29%	10% vs. 9%
Murthy et al.		RIC vs. MAC	2 years: 68% vs. 71%	2 years: 27% vs. 21%	2 years: 47% vs. 36%
MDS					
Kröger et al.	MDS/sAML	RIC vs. MAC	76% vs. 63%	17% vs. 25%	17% vs. 15%
Kurosawa et al.	MDS	RIC vs. MAC	47% vs. 45%	27% vs. 28%	30% vs. 29%
Jentzsch et al.	MDS	RIC vs. NMA	57% vs. 47%	24% vs. 38%	38% vs. 22%
Scott et al.	AML/MDS	RIC vs. MAC	68% vs. 78%	4% vs. 16%	48% vs. 14%
Craddock et al.	AML/MDS	seqRIC vs. RIC	61% vs. 59%	21% vs. 17%	27% vs. 30%
Potter et al.	MDS with blast excess	seqRIC vs. RIC vs. MAC	53% vs. 47% vs. 64%	23% vs. 35% vs. 23%	41% vs. 27% vs. 20%
Martino et al.	AML/MDS < 10%	RIC vs. MAC	53% vs. 56%	18% vs. 22%	34% vs. 24%

### 6.1 MDS

In MDS, RIC regimens appear to be associated with higher relapse risks but lower NRM, resulting in OS and PFS when compared to myeloablative conditioning (MAC), as shown in EBMT and CIBMTR studies. Prospective trials in AML and MDS (but predominantly AML) revealed a complex and inconclusive evidence landscape. For AML patients with intermediate‐/high‐risk cytogenetics and ≤ 60 years, or MDS/secondary AML patients, no significant differences in relapse or NRM were observed between conditioning regimens, though a trial by Kröger et al. suggested a trend towards better OS with RIC [70, 74]. Conversely, Scott et al. reported a reduced relapse risk and improved OS with MAC, despite its association with higher NRM in AML/MDS patients, with MDS patients being the minority and not evaluable for powered subgroup analysis [72, 75]. Attempts to intensify RIC through sequential RIC regimens did not yield superior outcomes compared to conventional RIC in high‐risk AML/MDS patients [71]. Meta‐analyses have been conducted to synthesize findings from these randomized trials, but their conclusions are limited by the heterogeneity of trial designs and patient characteristics [76]. Overall, the quality of evidence regarding the optimal conditioning intensity for MDS remains low.

In terms of the conditioning regimen, treosulfan appeared to be non‐inferior to busulfan and is particularly, beneficial as part of RIC regimens [77]. Total‐body irradiation has currently no role in conditioning regimens for MDS (as demonstrated most recently again) [78], and cyclophosphamide (outside of the PTCY platform) is not indicated in MAC regimens.

GVHD prophylaxis strategies remain debated, with PTCY increasingly utilized alongside the rise in haploidentical donors. A recent study from the EBMT analyzed 960 patients with MDS undergoing unrelated donor HCT with either PTCY or antithymocyte/anti‐T‐lymphocyte globulin (ATG) for GVHD prophylaxis. Neutrophil engraftment at day 28 was higher with ATG (93% vs. 85%); PTCY demonstrated superior outcomes with a 5‐year OS of 58% versus 49% for ATG and a PFS of 53% versus 44%. Acute GVHD (Grades 2–4) incidence was lower with PTCY (23%), while chronic GVHD incidence at 5 years was similar between the groups. Multivariable analysis confirmed better OS (hazard ratio, 1.32) and PFS (hazard ratio, 1.33) with PTCY. These findings may support a role for PTCY as a valid GVHD prophylaxis option for unrelated donor HCT, although main results may be applicable in the mismatched setting.

### 6.2 MF

Optimal conditioning intensity and regimen choice for HCT in MF remain challenging due to limited prospective trial data. Retrospective analyses, such as those from contemporary multicenter cohorts as well as historical data from the EBMT and CIBMTR, show comparable 5‐year OS between MAC and RIC, with MAC associated with higher relapse‐free survival but greater NRM [67, 68]. Fludarabine‐busulfan‐based regimens generally yield better outcomes in both MAC and RIC, while RIC is preferred for older or comorbid patients [69], and a triple combination of thiotepa, busulfan, and fludarabine is a frequent myeloablative regimen (at least in Europe) [79]. Prospective trials have highlighted age as a significant determinant of outcomes, with busulfan‐based RIC showing reduced mortality in older patients. Most recent studies showed that treosulfan (if given as RIC strategy) appears to be non‐inferior to RIC busulfan in MF and RIC busulfan is better than RIC melphalan [80, 81].

For GVHD prophylaxis, calcineurin inhibitors with methotrexate (or mycophenolate mofetil) and ATG are effective for matched‐sibling donors and MUDs, reducing acute and chronic GVHD [82, 83, 84]. PTCY has emerged as a viable alternative, particularly, for haploidentical donors, improving GVHD‐free and relapse‐free survival. Evidence supports PTCY over traditional ATG regimens for mismatched unrelated donors. A RIC‐based HCT combining cyclophosphamide, fludarabine, and total‐body irradiation, with PTCY, tacrolimus, and sirolimus as GVHD prophylaxis, demonstrated low rates of chronic GVHD in patients with MF undergoing transplantation from matched or mismatched related or unrelated donors. However, it was associated with a high 3‐year relapse rate of 40% [85].

The role of JAK inhibitors as part of the HCT protocol remains investigational. However, a recent study compared transplant outcomes in three groups: patients continuing JAK inhibition through conditioning to engraftment (PERI‐group), those stopping JAK inhibition at conditioning start (PRE‐group), and those never receiving JAK inhibition (NON‐group) [86]. The PERI‐group showed superior immune recovery, including early B‐cell and late gamma‐delta T‐cell and NK cell recovery, along with excellent engraftment rates (100% for neutrophils and platelets) and, importantly, no increased hematotoxicity or infections. Acute GVHD grade II‐IV incidence at day 100 was lowest in the PERI‐group (15%) compared to the PRE‐group (29%) and NON‐group (34%). The 1‐year relapse rate was also lowest in the PERI‐group (9%) compared to the PRE‐group (16%) and NON‐group (18%). These findings suggest peri‐transplant JAK inhibition is feasible and associated with favorable engraftment and GVHD outcomes, warranting further investigation.

In conclusion, conditioning intensity and GVHD prophylaxis should be adapted in view of individual patient and disease characteristics, with further studies needed to refine regimens, optimize GVHD management, and explore ruxolitinib's role in GVHD prophylaxis [87]. Both MAC and RIC are valid options, with conditioning intensity selected based on patient fitness, age, and disease risk. For haploidentical strategies, MAC‐based HCT might be preferred in light of relatively high relapse rates with RIC‐based approaches.

## 7 Pretreatment

### 7.1 MDS

In 2017, an expert panel recommended cytoreductive treatment before transplant conditioning in MDS patients with ≥ 10% bone marrow blasts [3], though evidence remained limited, and no prospective trials directly compared pre‐transplant therapy with no therapy. While pre‐transplant cytoreduction may offer benefits, there is no consensus on the optimal treatment type. Studies suggest that induction chemotherapy or HMAs do not consistently improve transplant outcomes, with the VidazaAllo study reporting disease progression or death from infections in some patients treated with azacytidine, preventing them from reaching HCT [28]. Conversely, the BMT‐AZA study demonstrated the feasibility of bridging high‐risk MDS to HCT [88].

A registry‐based EBMT study questioned the effectiveness of downstaging IPSS‐R risk with pre‐transplant therapy, highlighting the need for prospective trials. In particular, outcomes were unaffected by changes in IPSS‐R scores among untreated patients but were moderately better in those who received chemotherapy and achieved an improved IPSS‐R score at the time of HCT. Conversely, improvements in IPSS‐R scores following treatment with HMAs or other therapies did not demonstrate any beneficial effect on HCT outcomes. Notably, patients whose IPSS‐R scores worsened after receiving chemotherapy, HMAs, or other therapies had poorer HCT outcomes compared to those who received no prior treatment [89].

The ongoing ACROBAT trial aims to assess allo‐HCT feasibility in higher‐risk MDS, comparing upfront transplantation versus azacitidine or chemotherapy. Additional trials include a French study evaluating upfront allo‐HCT (NCT06235398) and the PALOMA trial, which is comparing Vyxeos to conventional care regimens before allo‐HCT.

### 7.2 MF

For MF, the main goal of treatment prior to HCT is aimed at reducing symptom burden, as HCT is the only real disease‐modifying treatment to date. To manage splenomegaly before HCT, options include splenic irradiation, splenectomy, and drug treatment. JAK inhibitors, particularly, ruxolitinib, are the mainstay before HCT to reduce spleen size and symptom burden, with the best outcomes reported in patients showing a sustained spleen response [39]. For thrombocytopenic patients, alternative inhibitors like pacritinib or fedratinib may be beneficial, while momelotinib offers additional advantages, such as improving hemoglobin and reducing transfusion dependence [90, 91, 92]. Patients with an ongoing spleen response to JAK inhibitors have better posttransplant outcomes, and a treatment duration of around 3 months before HCT is generally recommended. Non‐responders to ruxolitinib may be considered for second‐generation JAK inhibitors or investigational drugs, particularly, those at high risk according to MTSS.

**FIGURE 3:**
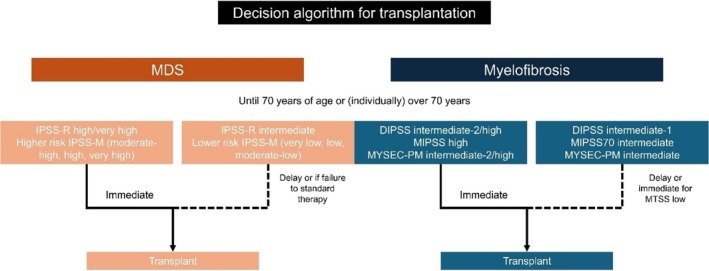
Simplified transplant decision algorithm for MDS and MPN.

Other strategies in refractory patients with splenomegaly include splenic irradiation or splenectomy. The only comparative study stems from a global initiative comparing splenectomy and splenic irradiation in JAK inhibitor refractory patients, to reduce spleen size [93]. This study showed significantly reduced relapse incidence with splenic irradiation in a very comorbid cohort with a spleen size of 23 cm median, without higher NRM and similar OS. The importance of using splenic irradiation may be the use directly as part of the HCT algorithm (even as part of the conditioning) to reduce potential hematotoxicity. This study also underscored that moderate‐dose irradiation (3–4 Gy) may suffice and achieve similar response rates. In contrast, splenectomy of very large spleens is complex and can delay HCT due to complications, and has been consistently shown to be associated with a higher risk of relapse [94, 95]. However, it may be an option for selected patients if planned appropriately, balancing the risk of such an intervention with the benefit of promising engraftment, shown in several studies [96, 97]. A decision algorithm for transplantation is shown in (Figure 3).

## 8 Poor Graft Function or Graft Failure

Cytopenia or pancytopenia posttransplant in MDS and MF can manifest as primary graft failure or poor graft function. There is limited evidence for strategies to reduce risk or treat established poor graft function [98], though the use of thrombopoietin analogs is emerging, albeit with limited data. CD34+ stem‐cell boosts have shown promise in HCT and novel cellular therapies [99], with studies reporting hematological improvement in 81% of patients, including those with MF, typically within 30 days of infusion [100]. These boosts are particularly, effective in HLA‐identical sibling transplants and can serve as definitive treatment in patients without active GVHD [101, 102].

For poor graft function, growth factors such as erythropoietin or granulocyte colony‐stimulating factor may be used as supportive measures, though they are unlikely to reverse the condition. Splenectomy might be an option in cases with persistent splenomegaly and full donor chimerism, but it carries risks. JAK2 inhibitors may help reduce spleen size and constitutional symptoms, but they lack evidence for treating poor graft function and may negatively affect hematopoiesis.

Early identification and management of primary graft failure (before Day 28) is needed, with rapid consideration of early donor recruitment being critical for timely intervention and decision for second transplant if necessary. Preventative strategies to prevent poor graft function include using HLA‐matched donors, high CD34+ cell doses, and controlling bulky splenomegaly before transplantation.

## 9 Molecular Monitoring After Transplantation

### 9.1 MDS

Chimerism analysis is a well‐established method for monitoring graft function and detecting disease recurrence following HCT [103]. Traditional techniques for assessing mixed chimerism include the use of sex‐chromosome‐specific probes (e.g., X and Y chromosomes) in fluorescent in situ hybridization studies and polymerase chain reaction (PCR) amplification of polymorphic short tandem repeat sequences. These methods offer sensitivity levels capable of detecting 1%–5% admixtures.

Recently, NGS assays have been introduced, offering enhanced sensitivity. In a study of 90 MDS patients who underwent HCT, persistent mutations with a variant allele frequency (VAF) of at least 0.5% were found in 37% of patients 30 days posttransplant [104]. Patients with these persistent mutations had significantly higher rates of disease progression (53% vs. 13%; *p* < 0.001) and lower 1‐year PFS (31.3% vs. 59.3%; *p* = 0.005) compared to those without detectable mutations. The impact was particularly, pronounced in patients receiving RIC, where persistent mutations were strongly associated with lower PFS. Multivariate analysis confirmed that a VAF of ≥ 0.5% at day 30 was independently linked to higher progression risk (hazard ratio 4.48, *p* < 0.001) and lower PFS (hazard ratio 2.39, *p* = 0.002). These findings highlight the prognostic value of early posttransplant mutation detection in predicting outcomes and guiding management for MDS patients undergoing HCT. Contemporary kits are even capable of identifying admixtures as low as 0.3%.

In another most recent prospective observational study in 266 MDS patients undergoing HCT, the 3‐year relapse‐free survival and OS rates were 59% and 64%, respectively. Measurable residual disease (MRD), assessed using NGS and droplet digital PCR, was available for 221 patients. Among 44 relapsed patients with complete MRD data, MRD status strongly influenced outcomes, with 1‐year relapse‐free survival rates decreasing as MRD levels increased: 49% (≥ 0.1%), 39% (≥ 0.3%), and 30% (≥ 0.5%). Multivariate analysis identified MRD positivity, WHO AML subtype, TP53 multihit mutations, NRAS mutations, and acute GVHD grade III‐IV as predictors of shorter relapse‐free survival. MRD positivity was also independently associated with shorter OS. Notably, in MRD‐positive patients, the presence of chronic GVHD was linked to improved outcomes.

In sum, MRD assessment is recommended every 3 months in the first year posttransplant and annually thereafter or as clinically indicated, with the potential to guide future interventions to reduce relapse.

### 9.2 MF

Driver mutations such as *JAK2, MPL*, and *CALR* are present in about 90% of MF patients and serve as key markers for monitoring MRD after HCT. Additional myeloid gene‐associated mutations, found in 40%–50% of patients through NGS, may be linked to the MF clone or independent clonal hematopoiesis. Recent evidence suggests that complete driver mutation clearance at day 30 after HCT is associated with improved relapse incidence, disease‐free survival, and OS. Mutation clearance appeared to outperform traditional chimerism analysis (which is useful but not as powerful as for MDS, given that patients due to extensive fibrosis usually need more time to acquire full donor chimerism). Achieving MRD negativity at days 100 and 180 post‐HCT appears to be associated with better outcomes compared to patients who did not achieve MRD negativity at any time. However, the survival and prognostic benefits were less pronounced than those observed in patients who attained MRD negativity as early as Day 30 after HCT. This suggests that while MRD negativity at later time points still confers an advantage, achieving it earlier in the posttransplant period is more strongly predictive of improved long‐term outcomes [105, 106].

Current consensus recommends molecular monitoring after HCT to enable timely immunotherapy interventions. Monitoring should occur at 1 month and every 3 months up to 1 year, with annual testing advised for detecting late relapses, which are often treatable with donor lymphocyte infusion (DLIs) or second transplants. High‐sensitivity assays, such as droplet digital PCR for *JAK2 V617F* (sensitivity < 0.01%), are preferred. Assays for *MPL* and *CALR* should achieve sensitivity < 1%. Peripheral blood is recommended over bone marrow for DNA analysis due to convenience, with no evidence supporting one source over the other.

Data on NGS after HCT do not exist, and using NGS in this setting is not currently recommended for MRD monitoring due to its limited sensitivity (< 1%) in routine settings. Assays for other mutations, such as *IDH1, IDH2*, and *DNMT3A*, remain investigational, although it has been shown that in the case of triple‐negative driver mutation genotype, measuring these mutations with high sensitivity might help guide monitoring in this population of unmet need [107]. MRD testing should be performed in accredited laboratories adhering to stringent quality control standards.

## 10 Relapse After Transplantation

Relapse remains an unmet clinical need in both MF and, more importantly, in MDS. We summarized current approaches that have been studied in both entities in Table 2 [108, 109, 110, 111, 112, 113, 114, 115].

**TABLE 2:** Selected approaches to relapse in MDS and myelofibrosis.

Study	Rationale	Outcome
Schroeder et al.	Azacytidine, lenalidomide and DLI for MDS/AML/CMML	Overall response: 56% Complete response: 50% Median OS: 21 months
Poire et al.	Azacytidine and DLI for AML/MDS	Overall response: 22% Complete response: 20% Median OS: 6 months
Yan et al.	AML/MDS and ALL; if MRD‐positive and no GVHD: DLI (plus IL‐2, partially with chemotherapy) or IL‐2 only	Improved OS and reduced relapse rate with DLI compared with IL‐2 only 3‐year OS: DLI, 58%; IL‐2 only, 28%
Miyamoto et al.	DLI with or without preceding chemotherapy for AML/MDS	Complete response in MDS for DLI: 30% Factors associated with response in entire group: GVHD, molecular/cytogenetic relapse compared with hematologic relapse
Gagelmann et al.	DLI for molecular or hematological relapsed MF	Complete response: 90% for molecular relapse, 60% for hematological relapse 5‐year OS: 80% vs. 32% Response after first DLI defines outcome
Ngo et al.	Hypomethylating agents (mostly decitabine)	Complete response: 50% Chronic GVHD: 50%
Klyuchnikov et al.	Salvage DLI and/or second HCT for MF	Complete response to DLI: 39% Overall response to HCT: 80% 2‐year OS: 70%
McLornan et al.	Outcome of relapsed MF receiving DLI, DLI and second HCT, or second HCT alone	Median OS: 76 months for DLI alone, 54 months for DLI + 2nd HCT, 27 months for 2nd HCT

### 10.1 MDS

Relapse remains a major problem after HCT for MDS. Approaches to reduce relapse include preemptive treatment for patients identified to be high‐risk pretransplant or who have persistent MRD‐positive status posttransplant. Various treatment approaches are available, including palliative care, adoptive immunotherapy with DLIs and/or tapering immunosuppression, HMA, and cellular immunotherapy. HMA‐based approaches have recently emerged to treat high‐risk and MRD‐positive patients. Ongoing clinical trials that include preemptive treatment of MDS patients post‐HCT are ongoing (NCT04078399, NCT06138587, NCT01541280, NCT04742634, NCT06492707, NCT05788679).

### 10.2 MF

The EBMT has established definitions for molecular, cytogenetic, and morphological or clinical relapse in MF posttransplant to guide management and standardize clinical practice [116]. DLI is a proven strategy for addressing relapse, particularly, when used preemptively at the stage of molecular relapse [113, 114, 117], where it induces higher rates of molecular remission (88%) compared to use during hematological relapse (60%) [113]. Additionally, preemptive DLI together with molecular monitoring has a lower risk of GVHD, with approximately half of patients achieving molecular remission without GVHD.

Taken together, preemptive DLI after cessation of immunosuppression is the standard of care for persistent MRD and should continue until complete remission or MRD clearance is achieved. DLI is also indicated for molecular or hematological relapse, but prophylactic DLI and the use of JAK inhibitors as maintenance therapy to prevent relapse are not currently supported by evidence.

As shown above for MDS, HMAs have demonstrated potential as salvage therapy for relapse posttransplant by inducing immune responses and improving donor chimerism. Data in MF are limited to a small single‐center cohort and one case report [110, 118]. A retrospective analysis of 12 patients with relapse showed that after two cycles of HMA (11 decitabine and one azacitine), 58% of patients showed restored donor chimerism. Half of the patients achieved driver molecular clearance, although one caveat of the study was the limited sensitivity of the NGS‐based kit of 0.3%. Chronic GVHD developed in 50% of patients, mostly mild to moderate, resolving after treatment. Another recent case report suggested disease control with decitabine, but this patient did not achieve complete chimerism until the last reported follow‐up, highlighting overall that this therapy should remain investigational.

## 11 Conclusion

Despite advancements in HCT for MDS and MF, several challenges and research avenues remain. Optimizing conditioning regimens, particularly, tailoring the intensity to individual patient profiles, requires further exploration through prospective trials. The role of MRD in guiding preemptive interventions before and after HCT is a promising area that needs standardization and broader implementation. New strategies to manage relapse warrant systematic investigation. Additionally, the integration of JAK inhibitors and novel targeted therapies into pre‐ and posttransplant care for MF holds potential for improving outcomes but requires more robust data. These efforts will be pivotal in improving survival, reducing relapse, and enhancing quality of life for individuals undergoing HCT.

And overall, it never gets old but is very true for both MDS and MF; we need prospective trials to account for selection bias and center differences to inform clinical practice for all.

## Author Contributions

N.G. and N.K. wrote the manuscript and approve of the final version.

## Ethics Statement

The authors have nothing to report.

## Consent

The authors have nothing to report.

## Conflicts of Interest

The authors declare no conflicts of interest.

## Data Availability

Original data can be retrieved from the corresponding author upon reasonable request.
